# Medicinal Plants for Major Depressive Disorder

**DOI:** 10.3390/brainsci16020223

**Published:** 2026-02-13

**Authors:** Amanda Gollo Bertollo, Luiza Spohr, Ana Élica Bearzi, Kelli Maria Kreuz, Zuleide Maria Ignácio

**Affiliations:** 1 Graduate Program in Neurosciences, Federal University of Santa Catarina, Florianópolis 88035-972, SC, Brazil; amanda.bertollo@estudante.uffs.edu.br (A.G.B.); ana.bearzi@posgrad.ufsc.br (A.É.B.); 2 Graduate Program in Biomedical Sciences, Laboratory of Physiology, Pharmacology, and Psychopathology, Federal University of Fronteira Sul, Chapecó 89815-899, SC, Brazil; luiza.spohr@estudante.uffs.edu.br (L.S.); kelli.kreuz@estudante.uffs.edu.br (K.M.K.)

**Keywords:** major depressive disorder, medicinal plants, neurotransmitters, neuroplasticity, neuroinflammation, HPA axis

## Abstract

**Highlights:**

**What are the main findings?**

- Medicinal plants modulate neurotransmitters, neuroplasticity, and the HPA axis to treat MDD.
- Some bioactive compounds reduce neuroinflammation and oxidative stress by inhibiting pro-inflammatory cytokines.

**What are the implications of the main findings?**

- Some plants offer promising therapeutic or adjunct alternatives for MDD.
- Clinical integration requires rigorous attention to safety regarding hepatotoxicity risks and significant herb–drug interactions.

**Abstract:**

Major Depressive Disorder (MDD) is a severe, chronic illness for which conventional treatments often show limited efficacy and side effects, driving a renewed interest in traditional medicinal plants. The therapeutic promise of these plants lies in their multi-targeted action, influencing neurotransmitter systems, modulating neuroinflammation and oxidative stress, impacting neuroplasticity, and regulating the Hypothalamic–Pituitary–Adrenal (HPA) axis. Despite their clinical potential, the use of medicinal plants is associated with challenges, including complex pharmacokinetics, significant adverse effects, and the risk of herb–drug interactions, alongside concerns regarding standardization and quality control. This manuscript aims to examine the therapeutic potential of key medicinal plants for managing MDD, including *Hypericum perforatum*, *Rhodiola rosea*, *Melissa officinalis*, *Passiflora incarnata*, *Valeriana officinalis*, and *Cannabis sativa*. Additionally, the review addresses emerging candidates such as *Curcuma longa*, *Withania somnifera*, *Panax ginseng* and *Centella asiatica*. By focusing on their mechanisms of action, pharmacokinetics, and associated risks, this review provides a more comprehensive understanding of their role in modern psychiatric care.

## 1. Introduction

Major Depressive Disorder (MDD) is a severe, chronic illness affecting millions worldwide. Despite the availability of conventional antidepressants, many patients do not achieve complete remission due to limited efficacy, treatment resistance, or intolerable side effects [1,2]. This has prompted a renewed interest in traditional and complementary therapies, particularly medicinal plants, which offer a promising alternative or adjunct to standard care. The use of these plants for mood disorders is deeply rooted in historical practice, and recent scientific efforts are now validating and elucidating their mechanisms of action [3].

The therapeutic potential of these plants lies in their ability to modulate multiple biological pathways associated with depression. Their active compounds can influence neurotransmitter systems, such as serotonin and dopamine, like conventional drugs [4]. Many of these plant-based compounds also possess anti-inflammatory and antioxidant properties, which are significant because chronic inflammation and oxidative stress are associated with the development of depression [5]. Furthermore, these plants can influence neuroplasticity, which is the brain’s ability to adapt and form new connections, and they can help regulate the body’s stress response system, specifically the hypothalamic–pituitary–adrenal (HPA) axis [6].

Despite their promising neuroprotective effects, some medicinal plants show some complicated challenges, including their interaction with drugs or uneven pharmacokinetic properties, in addition to some serious adverse effects [7]. For example, some plants can alter how the body processes other medications, thereby reducing their effectiveness or increasing their side effects [8]. The lack of standardized products and quality control in the market is also a concern, as it can affect both the potency and safety of these treatments [9,10]. This manuscript will examine the therapeutic potential of key medicinal plants for managing MDD, focusing on their mechanisms of action, pharmacokinetics, and associated risks, to provide a more complete understanding of their role in modern psychiatric care.

## 2. Main Medicinal Plants with Antidepressant Potential

Several medicinal plants have been investigated for their antidepressant potential, with promising results from preclinical and clinical studies. In this review, we present findings related to the medicinal plants *Hypericum perforatum*, *Rhodiola rosea*, *Melissa officinalis*, *Passiflora incarnata*, *Valeriana officinalis*, and *Cannabis sativa*. In addition, some emerging medicinal plants are also discussed.

### 2.1. Hypericum perforatum

*Hypericum perforatum* L., popularly known as St. John’s wort (SJW), is a species of the Hypericaceae family with a long history of use in traditional medicine systems. Its main bioactive constituents, such as hypericin, hyperforin, and flavonoids, confer to the plant a remarkable spectrum of therapeutic applications, particularly highlighting its antidepressant properties [11].

In the preclinical context, Kandilarov et al. (2018) evaluated plant extracts administered either in combination or separately in Swiss mice for eight weeks. Among the tested extracts, *Hypericum perforatum* (500 mg/kg, administered orally by gavage) demonstrated significant efficacy, as evidenced by a reduction in immobility time in the forced swim test (FST). The authors suggested that this antidepressant effect may be associated with the inhibition of serotonin, norepinephrine, dopamine, and glutamate reuptake [12].

Complementing this evidence, Bussmann et al. (2024) investigated the effect of the *Hypericum perforatum* Ze 117 extract (30, 90, and 180 mg/kg, p.o.) compared to escitalopram (10 mg/kg, p.o.) in rats subjected to corticosterone-induced depression. The study demonstrated that the 180 mg/kg dose of the extract, as well as escitalopram, attenuated depression-like behaviors, significantly increasing the latency to the first immobility in the FST [13].

In the clinical context, a double-blind trial evaluated the standardized *Hypericum perforatum* extract (WS® 5570) compared to paroxetine in patients with moderate depression. After 6 weeks of treatment, WS® 5570 showed greater reductions in Hamilton Depression Rating Scale (HAM-D) scores and higher response and remission rates. Adverse reactions were more frequent in the paroxetine group (61%) than in the *Hypericum perforatum* group (19%). The adverse effects associated with the plant were mainly gastrointestinal in nature, in addition to fatigue and dizziness [14]. While the results indicated that WS® 5570 was comparable in efficacy to other agents but had a more favorable safety profile, it is important to consider these findings within the broader clinical evidence. As noted in the Cochrane systematic review by Linde et al. [15], while many trials demonstrate robust efficacy, there is significant heterogeneity across studies worldwide. This variability is often attributed to differences in the chemical composition of commercial extracts, which complicates generalizing results across patient populations. Furthermore, while large-scale meta-analyses support its use in mild-to-moderate MDD, the evidence for severe depression remains less consistent, highlighting the need for further standardized, multicenter trials to establish definitive clinical guidelines.

In another 12-month controlled clinical trial, 60 participants with moderate depression received two commercial formulations of *Hypericum perforatum*: one conventional (Hp-C) and one multi-fractionated (Hp-MF). The treatment consisted of two daily tablets of Nervaxon® or IperiPlex®, both containing 300 mg of extract and 0.3% hypericin. Both treatments were well tolerated and showed no adverse effects. The results suggested that the Hp-MF extract may provide superior clinical outcomes compared to the extract obtained by Hp-C [16].

Finally, Xiang et al. (2025) conducted a randomized clinical trial with 198 participants with MDD and somatic complaints, comparing the ShuganJieyu capsule (a mixture of SJW and *Acanthopanax senticosus* L.) with SJW alone over eight weeks. The study indicated that the ShuganJieyu capsule showed efficacy and safety similar to SJW, with additional benefits observed in men and in patients with more severe somatic symptoms. Adverse effects were reported by 8% of participants receiving the ShuganJieyu capsule and by 6% of those receiving SJW, with no serious adverse effects reported [17].

### 2.2. Rhodiola rosea

*Rhodiola rosea* L. is an alpine perennial herbaceous plant and belongs to the Crassulaceae family. This plant is native to high-altitude regions, including East Asia, Central Asia, Siberia, and North America. It is also known as Golden Root, Arctic Root, and Roseroot [18].

Chen et al. (2009) investigated the effect of alcohol extract of *Rhodiola rosea* root (1.5, 3, and 6 g/kg) in animals exposed to chronic mild stress (CMS). This study demonstrated that the extract improved 5-hydroxytryptamine (5-HT) levels and induced neural stem cell proliferation in the hippocampus of depressive rats [19]. Corroborating previous data, study demonstrated that *Rhodiola rosea* extract (250 and 500 mg/kg) reversed depressive-like behavior induced by CMS in mice [20].

Yang et al. (2014) demonstrated the effect of salidroside, a bioactive compound present in standardized extracts of *Rhodiola rosea*. Salidroside (20 and 40 mg/kg for 2 weeks) alleviated olfactory bulbectomy (OBX)-induced hyperactivity and decreased immobility time in behavioral tests. Salidroside reduced tumor necrosis factor-alpha (TNF-α) and interleukin-1 beta (IL-1β) levels, increased glucocorticoid receptor (GR) and brain-derived neurotrophic factor (BDNF) expression in the hippocampus, attenuated corticotropin-releasing hormone (CRH) expression in the hypothalamus, and significantly reduced the levels of serum corticosterone [21].

Furthermore, Zhang et al. (2016) evaluated the effect of rhodioloside (20 and 40 mg/kg). Rhodioloside is a significant constituent of *Rhodiola rosea* roots, and the OBX model is a well-established animal model for evaluating the antidepressant activity of compounds. Rhodioloside significantly reversed OBX-induced reduction in sucrose consumption. In addition, rhodioloside decreased pro-inflammatory cytokines IL-1β and interleukin-6 (IL-6) levels and inhibited nuclear factor-kappa B (NF-κB) activation, as well as normalized the monoaminergic system changes in the prefrontal cortex of OBX rats [22].

Clinical studies of *Rhodiola rosea* have demonstrated antidepressant effects. A study demonstrated that *Rhodiola rosea* extract 340 mg (standardized to a content of rosavin 3.07%/rhodioloside 1.95%) administered over 12 weeks reduced HAM-D and Beck Depression Inventory (BDI) scores. However, the reductions were not statistically significant. Nonetheless, more patients reported adverse events using sertraline compared with *Rhodiola rosea* or placebo [23].

Furthermore, a survey by Gao et al. (2020) evaluated the antidepressant effects of a standardized *Rhodiola rosea* extract (0.3 and 0.6 g/day) compared with a placebo. This study demonstrated that *Rhodiola rosea* capsules showed antidepressant potential in patients with depressive disorder when administered at 0.3 or 0.6 g/day for 12 weeks, as indicated by reductions in HAM-D and BDI scores, as well as a change in Clinical Global Impression (CGI/C) scores. Regarding adverse effects, the study reported no serious adverse events. There were more reports of adverse effects associated with sertraline compared to *Rhodiola rosea* capsules [24].

### 2.3. Melissa officinalis

*Melissa officinalis* L., commonly known as lemon balm, belongs to the Lamiaceae family and has been widely used in various traditional medicine systems, particularly in European and Iranian medicine, for the treatment of numerous diseases. The plant contains volatile compounds, triterpenoids, phenolic acids, and flavonoids, which contribute to its pharmacological effects. Studies with crude extracts and isolated compounds of *Melissa officinalis* have demonstrated anxiolytic and antidepressant activities, as well as positive effects on mood, cognition, and memory [25].

In preclinical models, treatment with *Melissa officinalis* extract reduced depressive-like behaviors and increased serum BDNF levels at doses of 100 to 150 mg/kg [26]. The hydroalcoholic *Melissa officinalis* extract also showed antidepressant effects at doses of 75 to 150 mg/kg in a chronic stress-induced depression model [27]. In addition, Talebi et al. (2022) observed that doses of 350 and 550 mg/kg were the most effective in reducing depressive behaviors in mice treated with *Melissa officinalis* before reserpine-induced depression [28].

Continuing, a three-week clinical trial conducted with 100 healthy adults presenting moderate symptoms of depression, anxiety, and stress found that oral administration of 400 mg/day of a phospholipid *Melissa officinalis* extract (Relissa™) resulted in significant improvements in these scores, as well as benefits in sleep quality and overall well-being, without reports of serious adverse events [29]. Similar results were observed in an eight-week clinical trial evaluating the efficacy of *Melissa officinalis* (2 g/day) compared to fluoxetine (20 mg/day) in the treatment of mild to moderate depression. The results showed that *Melissa officinalis* was as effective as fluoxetine, with fewer adverse effects [30].

Finally, a recent study evaluated the efficacy of *Melissa officinalis* extract on symptoms of depression, anxiety, and sleep quality in patients with type 2 diabetes mellitus presenting depressive symptoms. Participants received 700 mg/day of *Melissa officinalis* extract for 12 weeks. At the end of the study, a statistically significant reduction in depression and anxiety scores was observed in the intervention groups, although no impact on sleep quality was noted. No serious adverse events were reported [31].

### 2.4. Passiflora incarnata

*Passiflora incarnata* L. is an evergreen vine that grows up to 6 m. It belongs to the *Passiflora* genus and Passifloraceae family, and is also known as passionflower, maypops or passion fruit. Most species of the *Passiflora* genus are found in Central and South America, with rare occurrences in North America, Southeast Asia, and Australia [32,33].

*Passiflora incarnata* is one of the herbal remedies used to alleviate the effects of stress. It is important to emphasize that chronic long-term stress is a pathological condition, which may lead to affective disorders, such as depression. Studies have indicated that *Passiflora incarnata* contains indole alkaloids, which have been used as antidepressants or provide key structures for their development [5,34,35,36].

Jafarpoor et al. (2014) evaluated the effect of hydroalcoholic extract of *Passiflora incarnata* (200, 400, and 800 mg/kg) in male mice. This research demonstrated that *Passiflora incarnata* extract, compared with the control group, significantly reduced the duration of immobility in the FST and the tail suspension test (TST) [37].

It is essential to highlight the antidepressant activity of the methanolic extract of *Passiflora foetida* leaves, which also belongs to the *Passiflora* genus and Passifloraceae family. Research by Santosh et al. (2010) demonstrated that *Passiflora foetida* leaf extract (100, 200, and 300 mg/kg), administered intraperitoneally, decreased the immobility time of mice in the TST and FST. The effects are comparable to those of standard drugs, i.e., fluoxetine (20 mg/kg) and imipramine (15 mg/kg) [38].

Research developed by Ayres et al. (2017) demonstrated that administration of *Passiflora edulis* fo. Aqueous extract (AE, 100–300 mg/kg, po), ethyl acetate (AcOEt, 25–50 mg/kg, po), and butanol (BuOH, 25–50 mg/kg, po) induced antidepressant-like effects in mice [39]. Corroborating with data, Alves et al. (2020) demonstrated the antidepressant-like activity of *Passiflora edulis* extract (50 and 100 mg/kg) as well as the extract-loaded nanoparticles (5 mg/kg) in mice using the FST, where the latter increased the potency of the former by 10-fold [40].

Interestingly, studies have demonstrated the antidepressant-like effects of chrysin (5,7-dihydroxyflavone), a flavonoid isolated from plants, such as *Passiflora incarnata* [41]. Research developed by Filho et al. (2016) reported that chrysin (5 and 20 mg/kg for 28 days) increased sucrose consumption and decreased immobility in the TST in female mice exposed to CMS [42].

Additionally, chrysin at 20 mg/kg for 14 days produced an antidepressant-like effect in the FST in male mice subjected to depressive-like behavior induced by OBX [43]. Another study demonstrated that chronic administration (28 days) of chrysin at 1, 5, 10, and 20 mg/kg produced antidepressant-like effects in male *Wistar* rats in the FST [44].

### 2.5. Valeriana officinalis

*Valeriana officinalis* L. belongs to the *genus Valeriana* and the family Caprifoliaceae, and is commonly known as “valerian”. It is a perennial herb primarily distributed in the northern temperate zones of North America, Europe, and Asia [45,46]. According to a review developed by Çelik and Kirmizibekmez (2025), pharmacological studies on extracts prepared mainly from the below-ground parts of *Valeriana* species have revealed diverse bioactivities [47].

A study by Kandilarov et al. (2018) demonstrated that *Valeriana officinalis* extract (500 mg/kg for 8 weeks) significantly reduced immobility time in the FST induced by a CMS model (8 weeks), demonstrating its depressive-like effect [12]. In another study using the same animal model to induce depressive-like behavior, authors showed that a combination of *Aloysia triphylla citrodora*, *Citrus aurantium*, *Echium amoneum*, *Lavandula angustifolia*, *Melissa officinalis*, *Salix aegyptiaca*, *Valeriana officinalis*, *Viola odorata*, and *Cinnamomum zeylanicum* (0.23 mL/mouse, orally daily for the last 4 weeks) decreased immobility time in FST and TST [48].

A recent study developed by Merkeb et al. (2025) evaluated the antidepressant efficacy of a gummy formulation of *Withania somnifera* and *Valeriana officinalis* in murine models using the FST. This research demonstrated that these gummies reduced immobility time and increased climbing activity compared to the control group, indicating a robust antidepressant effect [49].

Several studies have demonstrated the antidepressant-like effects of other plants belonging to the *Valerian* genus. Recent research has demonstrated that *Valerian jatamansi* can improve depressive-like behavior [50,51]. In addition, Choi et al. (2020) demonstrated that *Valeriana fauriei* extract has antidepressant-like activity against chronic restraint stress-induced depression [52].

Importantly, a study by Bao et al. (2024) evaluated the genotoxicity, 14-day acute oral toxicity, 90-day subchronic oral toxicity, and teratogenicity of the aqueous extract of *Valeriana officinalis* root. This research revealed that this extract does not exhibit genotoxicity [45].

### 2.6. Cannabis sativa

*Cannabis sativa* L. is one of the oldest plants cultivated in East Asia and is considered an annual dioecious plant. Humans have widely used it for centuries for various purposes. The bioactive components of *Cannabis sativa* L. can be classified into two main groups: cannabinoids and terpenes [53,54].

Several studies have demonstrated the effect of *Cannabis sativa* and its bioactive components on depression. Shin et al. (2024) evaluated the antidepressant-like and anti-inflammatory effects of *Cannabis sativa* inflorescence extract in a lipopolysaccharide (LPS)-induced neuroinflammation model. This research demonstrated that the extract (10, 20, and 30 mg/kg) administered 30 min before LPS significantly improved depressive-like behaviors and decreased inflammation [55].

Lucindo et al. (2025) evaluated the effect of cannabidiol (CBD), one of the cannabinoid compounds derived from *Cannabis sativa*. This research demonstrated that CBD (30 mg/kg) mitigated anhedonia and reduced immobility episodes in the TST in isolated mice [56]. Interestingly, Poudel et al., (2024) demonstrated that oral CBD administration (10 mg/kg, three times a week for four weeks) improved doxorubicin-induced anxiety and depression-like behaviors [57].

In addition, Ribeiro de Novais Júnior et al. (2024) evaluated the behavioral response to the administration for 7 days of 15 and 30 mg/kg of a CBD isolate and a full-spectrum CBD product in a depressive-like behavior model induced by LPS. These authors demonstrated that the full-spectrum CBD extract at both doses, but not the CBD isolate, reversed the LPS-induced depressive-like behavior in the FST [58].

A study developed by Pérez-Valenzuela et al. (2023) evaluated the combination of two primary constituents of *Cannabis sativa*, CBD and Δ-9-tetrahydrocannabinol (THC). These authors demonstrated that the consumption of CBD (30 mg/kg), THC (0.3 mg/kg), or a 1:100 combination of THC: CBD produces significant anxiolytic and antidepressant effects in stressed male rats [59]. Fang and Wang (2023) investigated the antidepressant-like effects of synthetic cannabinoid HU-210 (50 μg/kg). These authors demonstrated that HU-210 produces antidepressant-like effects in acute stress induced by forced swimming [60].

Ahn et al. (2021) investigated the anti-depressant effects of *Cannabis sativa* (hemp) seed ethanol extract (HE) in chlorpromazine (CPZ)-induced *Drosophila melanogaster* depression model. The experimental groups were treated with a single HE treatment (0.5%, 1.0%, and 1.5% of the media) and a mixture of CPZ and HE for 7 days. These authors demonstrated that HE administration alleviates depression-like symptoms through improving behavioral factors in the CPZ-induced *Drosophila* model [61].

Silote et al. (2021) investigated the effects of CBD in two different strains of mice (Swiss and C57BL/6) and a rat model of depression based on selective breeding (Flinders Sensitive and Resistant Lines) subjected to tests predictive of antidepressant-like effects. They investigated the influence of sex in the effects of CBD in both mice and rats. These authors observed that CBD induced an antidepressant-like effect, but strain, sex, and administration time affect CBD’s behavioral response to rodents [62].

Prospective and observational cohort study developed by Wolinsky et al. (2025) involved an assessment of the short and long-term effects of medicinal *Cannabis sativa* among individuals with clinically significant anxiety and/or depression newly starting medicinal *Cannabis sativa* use in Maryland, a state in the United States of America (USA) that allows medicinal use. In general, initiation of THC-dominant medicinal *Cannabis sativa* was associated with acute reductions in anxiety and depression, and sustained reductions in overall symptom severity over 6 months [63].

It is important to highlight that the guidelines recommend caution in using THC in patients with anxiety or mood disorders, since THC and CBD can have opposite effects on anxiety. Furthermore, responses to cannabinoid-based medications depend on the activity of the patient’s endocannabinoid system, the proportion of phytocannabinoids, the terpenoid composition, and the dose used [64]. Corroborating, systematic review and meta-analysis showed that TCH administration can induce positive, negative, and general psychiatric symptoms, with large effect sizes. However, CBD does not induce psychiatric symptoms [65].

Henderson et al. (2023) evaluated the potential for toxicity following repeated oral exposure to hemp-derived CBD isolate in rats. Dose levels of 0 (vehicle control), 30, 70, or 150 mg/kg-bw/d of CBD were administered once daily via oral gavage for 14 days. No adverse treatment-related effects were observed following up to 90 days of treatment with CBD at any dose level tested. The oral no-observed-adverse-effect level was therefore determined to be 150 and 140 mg/kg body weight/day in 14- and 90-day toxicity studies, respectively [66].

Furthermore, the literature reviews have demonstrated the relationship between *Cannabis sativa* and its bioactive compounds, as well as their potential for treating psychiatric disorders, such as depression [67,68,69].

### 2.7. Emerging Plants

Other plants have also demonstrated antidepressant effects. For example, *Curcuma longa* L., also known as turmeric, is widely used in traditional medicine and presents antidepressant properties mainly attributed to curcumin [70]. A study in a murine chronic stress model showed that nanocurcumin exerts effects similar to fluoxetine, improving depressive behaviors and increasing BDNF and serotonin levels [70]. Additionally, 80% ethanolic extract of *Curcuma longa* also mitigated depressive behaviors in mice, possibly through modulation of N-Methyl-D-Aspartate (NMDA) and 5-HT7A receptor-mediated pathways [71].

In a clinical study, Lopresti and Drummond (2017) investigated the effects of curcumin extract in individuals with MDD over 12 weeks. Participants received either a low dose (500 mg/day) or a high dose (1000 mg/day) of curcumin, or curcumin in combination with saffron (*Crocus sativus* L.). Both doses resulted in antidepressant effects, with no significant differences between them or with the addition of saffron, and the reported adverse events were mild [72]. Furthermore, Kanchanatawan et al. (2018) evaluated curcumin (500–1500 mg/day) as an adjunctive treatment in patients with MDD for 12 weeks, with follow-up at week 16. Curcumin use produced noticeable antidepressant effects from week 12, which were maintained after four weeks. The treatment demonstrated good safety and tolerability [73].

*Withania somnifera* L. (Ashwagandha) is also known for its anxiolytic and antidepressant properties. In animal models of chronic stress, root extract reduced anxious and depressive behaviors with efficacy similar to fluoxetine, modulating inflammatory and biochemical markers and preserving BDNF and serotonin levels [74]. In adolescent mice, Gokdemir et al. (2025) compared *Withania somnifera* with sertraline and observed antidepressant effects in both treatments, with *Withania somnifera* showing a greater reduction in pro-apoptotic proteins and inflammatory markers [75].

A randomized, double-blind, placebo-controlled clinical trial evaluated the effect of standardized root extract containing 2.5% withanolides (500 mg/day) with 5 mg of piperine for 90 days in individuals with mild to moderate depression and anxiety. The treatment was associated with a significant increase in serum serotonin levels, suggesting that monoaminergic neurotransmitters may mediate its antidepressant and anxiolytic effects [76].

*Panax ginseng* has also demonstrated antidepressant effects in animal stress models, acting mainly on the dopaminergic and serotonergic systems and modulating BDNF expression [77]. Additionally, it was observed to improve depressive behaviors by enhancing astrocytic gap junction function [78]. In a clinical context, patients with treatment-resistant MDD received *Panax ginseng* in capsules for six weeks, maintaining antidepressant monotherapy. The study indicated the purported efficacy and tolerability of *Panax ginseng* in clinical practice [79].

Another plant gaining notoriety is *Centella asiatica*, which, along with its bioactive compounds, has significant pharmacological potential for the treatment of MDD [80]. After being metabolized by the body, its compounds modulate neurotransmitters such as serotonin and dopamine [81]. In addition, a study in rodents found that chronic administration of the compound reduced depressive-like behaviors, such as anhedonia and immobility in the FST, and reduced oxidative stress [80].

While the discussed emerging plants show significant promise, a critical translational gap remains between preclinical observations and clinical application. The robust antidepressant-like effects observed in rodent models of *Curcuma longa* [70], *Withania somnifera* [74], and *Centella asiatica* [80] often rely on behavioral paradigms such as the FST and the TST. Although these tests are standard for screening compounds, they primarily measure behavioral despair or stress reactivity rather than the complex, multifaceted pathology of MDD in humans [15]. Consequently, high efficacy in murine models does not always translate to clinical success. Current clinical evidence for these plants, such as the studies by Lopresti and Drummond [72] on curcumin or Majeed et al. [76] on Ashwagandha, is often limited by small sample sizes and short follow-up periods. Further rigorous, long-term randomized controlled trials are essential to confirm whether the neurobiological mechanisms identified in animal models, such as BDNF modulation and HPA axis regulation, produce sustained symptomatic remission in patients with MDD [82].

## 3. Pharmacokinetics Regarding Medicinal Plants for Major Depressive Disorder

Medicinal plants are promising alternatives or adjuvants in the treatment of MDD. Parameters such as area under the plasma concentration curve (AUC), maximum concentration reached (Cmax), time to achieve it (Tmax), and elimination half-life (t½) allow us to estimate bioavailability and the duration of therapeutic action. In addition, many metabolites of medicinal plants are metabolized by cytochrome P450 (CYP) and uridine 5′-diphospho-glucuronosyltransferases (UGTs) enzymes, which can interact with conventional drugs by inducing or inhibiting these pathways [83].

*Hypericum perforatum* contains bioactive compounds such as hyperforin, hypericin, and pseudohypericin, as well as flavonoids such as quercetin and isorhamnetin [84]. A study conducted in humans using 900 mg of the plant’s dry extract found that hyperforin exhibited a high AUC and a relatively high Cmax, with Tmax around 4.5 h and a half-life of approximately 17.5 h after a single dose. These values were maintained in multiple doses for 14 days [85]. In addition, *Hypericum perforatum* has the characteristic of inducing CYP3A4 and P-glycoprotein (P-gp), an action attributed to hyperforin and, as a consequence, can reduce the AUC of several drugs metabolized by CYP3A4 and transported by P-gp [86].

*Rhodiola rosea* owes its properties mainly to its bioactive compounds, rosavin, salidroside, and tyrosol [87]. Studies in humans have reported that the bioavailability of salidroside is limited, with a half-life of elimination of 4–6 h. In contrast, oral absorption is relatively rapid, with a Tmax of 1–2 h [88]. It is metabolized in the liver by CYP450 enzymes, although data remain scarce [89]. A study with salidroside in mice found that the highest plasma concentration was detected in liver tissue 30 min after administration. After 1 h, the plasma concentration gradually decreased, and distribution in the liver and kidney tissues increased, indicating that salidroside may be excreted by the kidneys [90].

Research indicates that the phenolic compounds found in the plant *Melissa officinalis*, such as rosmarinic acid [91], are metabolized in the liver via glucuronidation and hepatic sulfation, with subsequent urinary excretion [92]. The bioavailability of rosmarinic acid is considered limited; however, intestinal absorption is rapid, with a Cmax occurring between 1 and 2 h after administration [93].

*Passiflora incarnata*, in turn, contains flavonoids, alkaloids, and glycosides that act directly on the central nervous system (CNS) [94]. Research indicates that in vitro metabolism studies show that C-glycoside flavonoids are absorbed in the gastrointestinal tract, with a Tmax of 1–2 h after intestinal metabolism, and undergo biotransformation by conjugation via UGTs and sulfotransferases [95].

The *Valeriana officinalis* plant contains bioactive compounds, including valerenic acid and valepotriates (esterified iridoids) [96]. Some studies have shown that the plant has moderate oral absorption and extensive hepatic metabolism, which can affect CYP450 enzymes [97]. Research indicates that valerenic acid reaches a Cmax after nighttime oral administration, with a subjective onset for each individual, and has a half-life of elimination, suggesting that it does not accumulate excessively with repeated doses [97]. In addition, another study found that after oral administration of 600 mg of valerenic acid extract, Tmax occurred between 1 and 2 h, and its half-life was approximately 5–6 h [98].

*Cannabis sativa* is a plant gaining relevance because its main components are the phytocannabinoids THC and CBD, whose pharmacokinetic profiles vary depending on the route of administration and dosage. Both are highly lipophilic, have low oral bioavailability, and are extensively distributed in lipid-rich tissues, with large volumes of distribution and rapid CNS penetration after inhalation or oral administration [99].

CYP2C9 and CYP3A4 metabolize THC and have a short plasma half-life, while CBD is metabolized by CYP2C19 and CYP3A4, forming 7-hydroxycannabidiol (7-OH-CBD) and 7-carboxycannabidiol (7-COOH-CBD), with variable plasma t½ [100]. Other studies cite that cannabinoids administered by inhalation have similarities to those administered intravenously [101]. It is estimated that the half-life of THC varies between an initial 6 min and a terminal half-life of 22 h [102], while CBD has an average half-life after daily oral administration ranging from 2 to 5 days [103].

Some plants have become the subject of study for their therapeutic potential, namely *Panax ginseng, Centella asiatica, Curcuma longa,* and *Withania somnifera*, which are showing promising results in research [80,104,105].

The *Panax ginseng* plant contains ginsenosides as a bioactive compound. These triterpenoid saponins have low oral bioavailability and are metabolized by the intestinal and liver systems. After administration, the Tmax time is 1 to 4 h, with a half-life of 7 to 15 h, depending on the drug formulation. Hepatic metabolism occurs through deglycosylation and oxidation via CYP450 enzymes [106].

*Centella asiatica*, for example, is a plant that contains active compounds such as asiaticoside, madecassoside, asiatic acid, and madecassic acid [107]. Studies indicate that its compounds have low oral bioavailability due to their high polarity and hepatic metabolism [108]. A preclinical study found that asiaticoside can be distributed widely across tissues by binding to albumin [109]. Although its bioavailability is low, the bioactive compounds can be detected in feces 48 h after oral administration of *Centella asiatica* [110], suggesting that triterpene glycosides are metabolized in the intestine [108].

Another emerging plant is *Curcuma longa*, which has a short half-life in the gastrointestinal tract [111] and is metabolized in the liver, intestine, and intestinal microbiota [112]. When administered orally, it is metabolized into glucuronates and glucuronate/sulfate conjugates [113]. Other enzymes responsible for its metabolism are nicotinamide adenine dinucleotide phosphate (NADPH)-dependent reductase and alcohol dehydrogenase [114].

*Withania somnifera’s* main bioactive compound is withanolides, such as withaferin A. Studies in animal models have shown that withaferin A has an oral bioavailability of 32.4% and undergoes hepatic metabolism, with a short plasma half-life [115]. Furthermore, it is not a P-gp substrate, which may facilitate its intestinal absorption. Additionally, strategies such as the use of nanoparticles and co-administration with piperine enhance its absorption [116]. A human study found that oral absorption is variable and that withanolides are rapidly eliminated from the body. However, standardized formulations of the herbal medicine are needed to improve exposure to withanolides, as measured by ultra-high-pressure liquid chromatography with mass spectrometry (UHPLC-MS/MS) [117].

The pharmacokinetic profiles of these plants provide a critical biological explanation for the dosing regimens and varying success rates observed in clinical trials. A primary example is curcumin from *Curcuma longa*; despite its robust multi-targeted antidepressant potential [70], its therapeutic utility is severely limited by poor oral bioavailability, rapid metabolism, and a short half-life in the gastrointestinal tract [111,112]. This explains why clinical studies, such as those by Kanchanatawan et al. [73], necessitate high daily doses of up to 1500 mg to achieve noticeable antidepressant effects. Similarly, the high polarity of triterpenes in *Centella asiatica* results in low intrinsic bioavailability [108], which may account for the lack of robust clinical evidence in humans despite strong preclinical results [80]. These pharmacokinetic barriers underscore why the development of advanced delivery systems, such as nanocurcumin [70] or phospholipid carriers for *Melissa officinalis* [29], is not merely a technological improvement but a clinical necessity to achieve therapeutic concentrations in the brain and ensure antidepressant efficacy.

**Figure 1:**
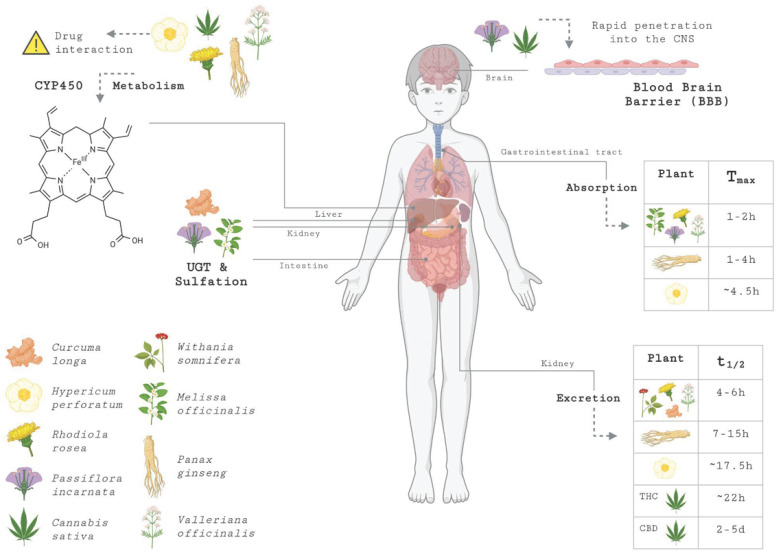
Pharmacokinetics of medicinal plants. The diagram illustrates the stages of absorption, distribution, metabolism, and excretion of the studied species. In absorption, the tables indicate the Tmax, highlighting the rapid absorption (1–2 h) of plants such as *Melissa officinalis* and *Passiflora incarnata*. In distribution, arrows indicate penetration into the CNS through the BBB. Metabolism is divided into Phase I (via CYP450) and Phase II (via UGT and sulfation); the warning icon specifically highlights the risk of drug interactions, notably for *Hypericum perforatum* due to its role as a potent enzymatic inducer. Excretion is represented by the t½, reflecting the duration of the compounds in the system before renal or fecal elimination. Central nervous system (CNS), blood–brain barrier (BBB), time required to achieve maximum concentration (Tmax), elimination half-life (t½), UDP-glucuronosyltransferase (UGT), cytochrome P450 (CYP450). Created in BioRender.

The pharmacokinetics of the aforementioned medicinal plants are illustrated in Figure 1.

Pharmacokinetic parameters vary considerably depending on the experimental model, formulation, dose and method. Differences in pharmacokinetic profiles were observed between isolated compounds and extracts, suggesting that phytochemical interactions may influence absorption, metabolism, and bioavailability. Table 1 summarizes pharmacokinetic parameters, including Cmax, Tmax, and elimination half-life, for compounds and extracts from medicinal plants.

## 4. Elucidating Neurobiological Mechanisms of Action

The therapeutic effects of medicinal plants in treating MDD are linked to their ability to modulate various neurobiological pathways. These plants contain a rich variety of bioactive compounds, including alkaloids, flavonoids, and terpenoids, that can influence key brain systems. Their mechanisms of action are often multi-targeted, which may explain why they can be effective in some cases where conventional antidepressants fail.

A fundamental requirement for the antidepressant efficacy of these plants is the ability of their bioactive constituents to cross the blood–brain barrier (BBB). Highly lipophilic compounds, such as the phytocannabinoids THC and CBD, cross the BBB rapidly after administration and are extensively distributed in lipid-rich tissues [99]. Other small molecules, such as valerenic acid [98] and various phenolic compounds, such as rosmarinic acid [89], also demonstrate central nervous system penetration and interact with GABAergic and monoaminergic targets [96]. In contrast, large polar molecules like asiaticoside [110] and ginsenosides [106] exhibit low intrinsic oral bioavailability and often require biotransformation by the intestinal microbiota into smaller, more lipophilic metabolites to exert central effects [108]. Advanced delivery strategies, such as nanoformulations [40,70] or co-administration with piperine [116], are essential tools for overcoming these permeability barriers and enhancing the neuroprotective potential of these plants.

**Figure 2:**
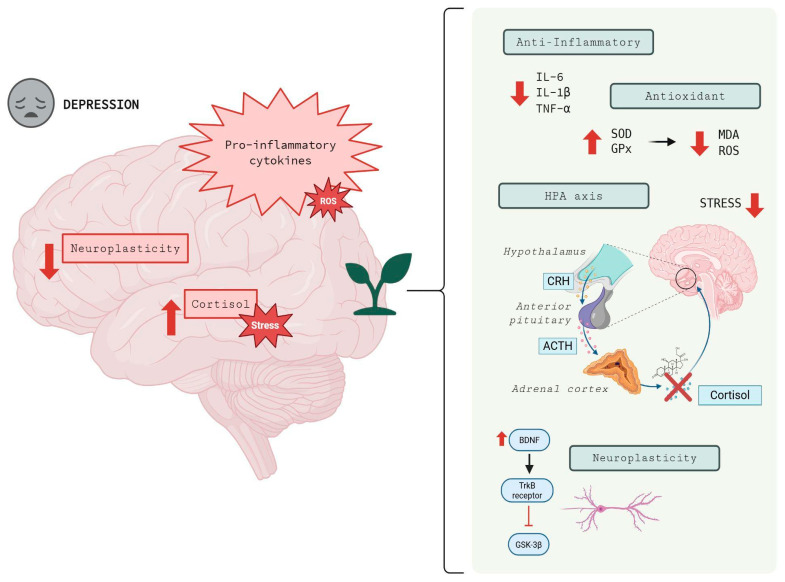
Anti-inflammatory, antioxidant and neuroprotective mechanisms of medicinal plants in MDD. The left section of the diagram illustrates the pathophysiological state of depression, characterized by neuroinflammation (pro-inflammatory cytokines), oxidative stress, HPA axis dysregulation (elevated cortisol and stress levels), and impaired neuroplasticity. The right panel demonstrates the therapeutic actions of medicinal plants: (1) Anti-inflammatory and antioxidant: downward arrows represent the reduction in pro-inflammatory cytokines (IL-6, IL-1β, TNF-α) and oxidative markers (ROS, MDA), while upward red arrows indicate the stimulation of antioxidant enzymes (SOD, GPx). (2) HPA axis modulation: medicinal plants attenuate the stress response (downward arrow) by regulating the secretion of CRH and ACTH, and inhibiting cortisol overproduction (indicated by the red “X”). (3) Restoration of neuroplasticity: bioactive compounds stimulate the BDNF/TrkB signaling pathway. Specifically, the red T-bar symbol represents the direct inhibition of GSK-3β, an enzyme whose suppression is crucial for promoting neuronal survival and synaptic plasticity. Major depressive disorder (MDD), hypothalamic–pituitary–adrenal (HPA), reactive oxygen species (ROS), interleukin-6 (IL-6), interleukin-1 beta (IL-1β), tumor necrosis factor-alpha (TNF-α), superoxide dismutase (SOD), glutathione peroxidase (GPx), malondialdehyde (MDA), corticotropin-releasing hormone (CRH), adrenocorticotropic hormone (ACTH), brain-derived neurotrophic factor (BDNF), tropomyosin receptor kinase B (TrkB), glycogen synthase kinase-3beta (GSK-3β). Created in BioRender.

The following sections will detail how these plants interact with neurotransmitter systems, modulate neuroinflammation and oxidative stress, impact neuroplasticity, and regulate the HPA axis (Table 2; Figure 2).

### 4.1. Interaction with Neurotransmitter Systems

Many medicinal plants exert their antidepressant effects by modulating the brain’s neurotransmitter systems, which are often dysregulated in MDD [4]. Similar to conventional antidepressants like selective serotonin reuptake inhibitors (SSRIs), some plants act by inhibiting the reuptake of key monoamine neurotransmitters, including serotonin, norepinephrine, and dopamine.

For example, *Hypericum perforatum* is known to inhibit the reuptake of serotonin, norepinephrine, dopamine, and even glutamate. This increases the concentration of these neurotransmitters in the synaptic cleft, improving mood and other depressive symptoms. The mechanisms of action for *Hypericum perforatum* are primarily attributed to its bioactive constituents, including hypericin, hyperforin, and flavonoids [11]. *Hypericum perforatum* also inhibits glutamate reuptake, contributing to its antidepressant effects [12].

Another way some plants modulate monoamines is by inhibiting monoamine oxidase (MAO), which breaks down neurotransmitters such as serotonin and dopamine [126]. This leads to increased levels of these neurotransmitters in the brain. Curcumin, a primary active compound from *Curcuma longa*, has been shown to inhibit both MAO-A and MAO-B enzymes in mice, contributing to its antidepressant effects [127]. Curcumin also interacts with serotonin receptors 5-HT1A/1B and 5-HT2C, and its antidepressant effects may be linked to an increase in the expression of the 5-HT1A receptor [128]. Curcumin has been observed to elevate norepinephrine and dopamine levels in various brain regions of rats, including the hippocampus [129].

Some plant compounds directly interact with neurotransmitter receptors. For example, valerenic acid from *Valeriana officinalis* is believed to enhance the effects of gamma-aminobutyric acid (GABA) by acting on its receptors. This modulation of the inhibitory GABA system may contribute to its anxiolytic and mild sedative properties, which can indirectly help with depressive symptoms [130].

Alterations in the GABA and glutamate systems also contribute to the pathophysiology of MDD, and some plant compounds can modulate these systems. GABA is the primary inhibitory neurotransmitter in the CNS, and its dysfunction is linked to mood disorders. Conversely, glutamate is the primary excitatory neurotransmitter, and its overactivity can lead to excitotoxicity and neuronal damage. By modulating these systems, medicinal plants can help restore the balance between excitatory and inhibitory signaling in the brain [131]. *Withania somnifera* (Ashwagandha) is believed to increase brain GABA levels, which may explain its anxiolytic and antidepressant properties [132].

Beyond serotonin and GABA, certain plants have been shown to significantly affect the dopamine and norepinephrine systems. Studies indicate that *Panax ginseng* can regulate monoamine neurotransmitters such as dopamine and norepinephrine, contributing to its antidepressant-like effects. Ginsenoside Rb3, in particular, exerts antidepressant effects through modulation of noradrenergic pathways [133]; and ginsenoside Rb1 enhances serotonergic neurotransmission [134].

The therapeutic effects of several plants are also linked to their influence on neurotransmitter systems. *Rhodiola rosea* extract has been shown not only to improve 5-HT levels in the hippocampus of depressive rats and normalize the monoaminergic system [19], but also in clinical settings: a six-week randomized placebo-controlled trial of the SHR-5 extract (340–680 mg/day) in humans with mild to moderate depression demonstrated significant improvements in overall depression, insomnia, emotional instability, and somatization [135], and another study combining *Rhodiola rosea* with sertraline showed greater reduction in depressive symptoms than with sertraline alone [24]. In animal models, *Rhodiola rosea* has also been found to reduce glutamate dysregulation, indicating modulation beyond just monoamines [136].

*Melissa officinalis* has been studied for its antidepressant-like and anxiolytic effects. A water extract reduced depressive-like behavior in rats and was associated with the modulation of serotonergic neurotransmission, including a decrease in serotonin turnover rate [137]. Components such as rosmarinic acid and certain flavonoids (e.g., quercitrin, luteolin derivatives) may contribute to the effects via MAO inhibition and serotonergic actions. Moreover, GABAergic activity has been implicated: rosmarinic acid can inhibit GABA transaminase (thus reducing GABA degradation) and bind to GABAA receptors, enhancing inhibitory signaling [138]. *Melissa officinalis* may also interact with cholinergic receptors and reduce corticosterone levels, possibly contributing to mood regulation [29].

*Passiflora incarnata* is another herb frequently used in mood disorders and anxiety contexts. Studies indicate that a dry extract modulates the GABA system by interacting with GABAA and GABAB receptors in humans, potentially facilitating the tapering of benzodiazepines [139]. *Passiflora incarnata* modulates not only the GABA system but also monoaminergic pathways (serotonin, dopamine, and norepinephrine) and the glutamatergic system (e.g., NMDA receptor inhibition), thereby exerting neuroprotective effects [140]. Administration of *Passiflora incarnata* extract altered neurotransmitter levels (especially dopaminergic and noradrenergic) in CNS structures, showing effects consistent with modulation of GABAergic signaling [141].

*Cannabis sativa*, especially CBD, has also been explored in depression. Preclinical studies demonstrate that CBD exhibits antidepressant-like behavior and modulates serotonergic transmission, as evidenced by its effect on 5-HT neurotransmission [142]. The antidepressant-like effect and the enhanced cortical 5-HT/glutamate neurotransmission induced by CBD were blocked by 5-HT1A receptor antagonism, suggesting a crucial role for that receptor [143]. CBD also reverses dysfunction in the excitatory/inhibitory balance, modulating glutamate and GABA signaling, in addition to effects on monoamines [144]. Deficits in endocannabinoid system components are linked with depressive phenotypes, and cannabinoids acting on CB1/CB2 receptors and enzymes like fatty acid amide hydrolase/monoacylglycerol lipase (FAAH/MAGL) may influence mood by altering neurotransmission and stress regulation [67].

**Figure 3:**
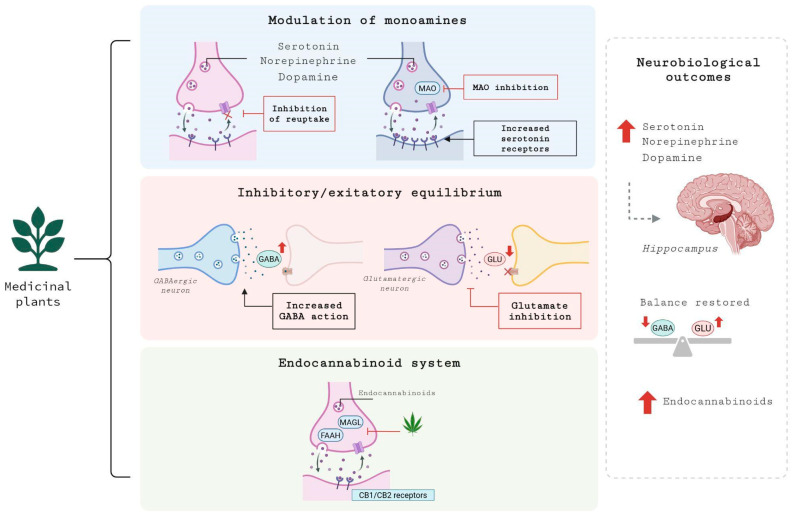
Mechanisms of action of medicinal plants on neurotransmitter systems in MDD. The diagram illustrates the action of bioactive compounds across three primary systems: (1) Modulation of monoamines, where plants inhibit the reuptake of serotonin, norepinephrine, and dopamine, in addition to inhibiting the MAO enzyme and increasing the expression of serotonin receptors, elevating the availability of these neurotransmitters in the synaptic cleft; (2) Inhibitory/excitatory balance, demonstrating the potentiation of the GABAergic pathway and the inhibition of glutamatergic signaling, which restores synaptic homeostasis in critical regions such as the hippocampus; and (3) Endocannabinoid system, in which the inhibition of the degradation enzymes FAAH and MAGL occurs, resulting in increased levels of endocannabinoids and their interaction with CB1/CB2 receptors. The neurobiological outcomes are indicated in the right column. Major depressive disorder (MDD), monoamine oxidase (MAO), gamma-aminobutyric acid (GABA), glutamate (GLU), fatty acid amide hydrolase (FAAH), monoacylglycerol lipase (MAGL), cannabinoid receptor type 1 and type 2 (CB1/CB2). Created in BioRender.

In summary, numerous medicinal plants exert antidepressant effects through diverse mechanisms that involve modulating the brain’s neurotransmitter systems. These actions include the inhibition of monoamine reuptake, inhibition of the MAO enzyme, direct interaction with neurotransmitter receptors, and regulation of the excitatory/inhibitory balance via GABA and glutamate pathways. Beyond these standard mechanisms, many plants, such as *Rhodiola rosea*, *Melissa officinalis*, *Passiflora incarnata*, and *Cannabis sativa,* also engage multiple neurobiological pathways, influencing neuroplasticity, neuroinflammation, and stress regulation. The effects of medicinal plants on the brain’s neurotransmitter systems are illustrated in Figure 3.

### 4.2. Anti-Inflammatory and Antioxidant Properties

The biological mechanisms involved in the actions that medicinal plants exert on anti-inflammatory and antioxidant pathways converge with the inhibition of IL-6/TNF-α, suppression of the NOD-like receptor family pyrin domain containing 3 (NLRP3) inflammasome, modulation of mitogen-activated protein kinase (MAPK), and activation of pathways that induce the release and expression of antioxidant enzymes [145]. Medicinal plants have been investigated because, in addition to helping in the treatment of depression, they act on the CNS by preventing neuroinflammation in limbic structures [146].

*Hypericum perforatum* is utilized as an herbal medicine due to its anti-inflammatory properties [147]. In animal studies, standardized extracts of the plant reduced malondialdehyde (MDA) and TNF-α, while increasing the expression of antioxidant enzymes [148]. Other data indicate that administering the compound has an anti-inflammatory effect on pro-inflammatory cytokine levels in animals, as evidenced by a decrease in IL-6, IL-1β, and TNF-α cytokines, suggesting its effectiveness in modulating neuroinflammation [149].

*Rhodiola rosea* demonstrated in chronic stress models that its extracts suppressed IL-6 and TNF-α expression, in addition to increasing the expression of antioxidant factors via nuclear factor erythroid 2-related factor 2 (Nrf2), which favored an increase in BDNF levels and decreased antidepressant behaviors in these animals [20]. In addition, research has shown that the expression of pro-inflammatory cytokines, TNF-α, IL-1β, and IL-6, was reduced by treatment with *Rhodiola rosea* constituents [150].

The *Melissa officinalis* plant exhibits antioxidant and anti-inflammatory properties [151], which have been studied in both in vitro and in vivo research on pathologies related to oxidative stress [152]. In animal studies, the plant’s bioactive compounds have been shown to reduce IL-1β, IL-6, and TNF-α, in addition to inhibiting microglial activation and increasing the activity of superoxide dismutase (SOD) and glutathione peroxidase (GPx) [153]. Furthermore, aqueous and ethanolic extracts of the plant increase the activity of antioxidant enzymes in animal models [154].

In turn, *Passiflora incarnata* contains bioactive compounds, including flavonoid C-glycosides and β-carboline alkaloids, which are responsible for its antioxidant and anti-inflammatory effects [155]. Research has shown that flavonoids reduce the production of reactive oxygen species (ROS) and MDA, which are related to oxidative damage. Additionally, in animal model studies, the plant demonstrated a reduction in IL-6 and TNF-α [95]. In vitro studies conducted with Wistar rats, using the administration of vitexin and isovitexin compounds by gavage, showed an increase in antioxidant capacity [156].

The *Valeriana officinalis* plant also exhibits antioxidant and anti-inflammatory effects, which are attributed to the compounds valepotriates and valerenic acid [157]. In addition, research conducted on mice with Parkinson’s disease has shown that the plant’s components have anti-inflammatory properties [158], and its antioxidant potential has been cited; however, the mechanisms underlying these effects have not yet been fully elucidated [159].

In turn, the *Cannabis sativa* plant has antioxidant activity related to its phytocannabinoids, mainly CBD. Its use in in vitro research with animal models has been linked to microglial inhibition and reduction in IL-1β and TNF-α, which reduces oxidative stress and inflammation [123,160]. CBD acts to interrupt the chain reactions of free radicals, transforming them into less active forms [161]. Additionally, it reduces oxidative stress conditions, as demonstrated in animal models of renal nephropathy treated with cisplatin [162].

Among emerging plants, *Panax ginseng, Withania somnifera, Centella asiatica*, and *Curcuma longa* also show promising anti-inflammatory and antioxidant profiles. Extracts from the root of the *Panax ginseng* plant have anti-inflammatory and antioxidant effects [163,164]. Additionally, it can modulate the expression of pro-inflammatory cytokines IL-6 and TNF-α [165]. *Withania somnifera* has demonstrated antioxidant potential in in vitro research using animal models and in clinical trials involving healthy individuals [166,167]. Its high levels of flavonoids can repair oxidative damage in cells, in addition to combating the formation of ROS [168,169].

*Centella asiatica* acts to modulate cytokines and increase the antioxidant enzymes SOD and GPx [170]. In research with animal models, its administration increased antioxidant activity in the brains and livers of animals [171]. *Curcuma longa*, in turn, activates the Nrf2/heme oxygenase-1 (HO-1) signaling pathway, acting to protect cells against oxidative stress. To increase its antioxidant effects, its administration has been optimized with piperine and nanoformulations [172].

### 4.3. Impact on Neuroplasticity and Cell Signaling

Neuroplasticity is marked by adaptive changes in the CNS involving trophic cascades and intracellular signaling, and the restoration of neuroplasticity is therefore a central mechanistic target in MDD [173]. *Hypericum perforatum* promotes neuroplasticity in preclinical models by stimulating tropomyosin receptor kinase B (TrkB)-dependent signaling and hippocampal neurogenesis, increasing synaptic markers such as postsynaptic density protein 95 (PSD-95) and synaptophysin (SYP) [174]. Hyperforin, one of the plant’s bioactive compounds, has been shown in animal studies to stimulate cAMP response element-binding protein (CREB) phosphorylation, suggesting a direct impact on neurotrophic signaling pathways and neuronal plasticity [175].

*Rhodiola rosea* acts on neuroplasticity through antioxidant and anti-inflammatory mechanisms, restoring and increasing BDNF expression, which promotes neurite growth and increases the phosphorylation of glycogen synthase kinase-3beta (GSK-3β) (an inhibitory kinase) and protein kinase B (Akt) [136]. The significance of GSK-3β in MDD lies in its role as a molecular hub that connects numerous signaling pathways essential for cellular homeostasis. While increased activity of this kinase in the PFC has been associated with MDD pathogenesis and the reduction in synapse size and neuronal excitability, its inhibition, facilitated by the BDNF/Akt pathway, is fundamental for the induction of long-term potentiation (LTP) and neuronal survival [176]. In animal models, *Rhodiola rosea* has also been found to activate the BDNF/TrkB-GSK-3β signaling pathway [136]. Limited studies in humans indicate modulation of stress reactivity and possible effects on cortical plasticity, but core molecular data are still scarce [177].

One study found that *Melissa officinalis* affects cognitive processes through the modulation of BDNF via compounds found in the plant [178]. Rosmarinic acid, one of the plant’s phytopharmaceuticals, increases BDNF expression and maintains synaptic proteins under stressful conditions, thereby helping to preserve the quantity and functionality of proteins essential for neuronal communication [179].

*Passiflora incarnata* acts by influencing neuroplasticity, primarily through the GABAergic pathway, thereby reducing neuronal hyperexcitability and attenuating stress-induced microglial activation [180]. The plant’s flavonoids increase antioxidant enzyme activity, reduce ROS and MDA, and indirectly support BDNF expression and synaptic stability in rodent models [181].

The plant *Valeriana officinalis* has been shown in in vitro research to modulate neuroplasticity with hippocampal neuron cultures, where it exerted effects against beta-amyloid (Aβ) protein toxicity by reducing excessive Ca^2+^ flux and lipid peroxidation, suggesting the regulation of intracellular calcium and oxidation as part of the neuroprotective effect [182]. Another study highlighted that administering the plant’s root extract modulated cortical excitability, thereby decreasing hyperexcitability in the cortex, which suggests an impact on intracortical facilitation and inhibition circuits and, consequently, an influence on synaptic plasticity [183].

*Cannabis sativa* performs its neuroprotective function through CBD, as it is a potent inhibitor of oxidative and nitrosative stress and also acts to reduce neuronal damage caused by increased Aβ protein deposition [184]. Furthermore, it attenuates the depletion of tyrosine hydroxylase levels, modulating ROS production. CBD also attenuated the expression of mitochondrial superoxide and intercellular adhesion molecules-1 (ICAM-1) and vascular cell adhesion molecule-1 (VCAM-1), suggesting that CBD may exert neuroprotective effects independent of CB1 and CB2 [185].

The root of the *Panax ginseng* plant and its bioactive compounds exhibit significant potential in neuroplasticity. Some studies have shown that these compounds activate neuronal survival cascades, in addition to activating TrkB receptors, which aid in the formation and elongation of neurites and dendritic spines [186]. Furthermore, one of the plant compounds cited in the scientific literature as having a neuroprotective role is Rb1, which acts by suppressing the expression of free radicals and increasing the stability of the mitochondrial membrane, thereby enhancing cell viability and protecting cells [187].

*Curcuma longa* has a positive effect on neuroplasticity. Some studies suggest that the plant can modulate neuronal plasticity pathways, including those involving CREB and BDNF. Additionally, an analysis performed by Western blotting revealed that curcumin stimulated BDNF synthesis [188]. Another study, conducted in mice using normobaric cerebral hypoxia models and treated with curcumin by gavage for 7 days, showed recovery of cognitive deficits and an effect on the emergence of hippocampal neurons [189].

*Withania somnifera* has positive effects on neuronal plasticity by increasing BDNF. In addition, in vitro studies with rodents have shown an increase in BDNF protein, greater neurite growth, and a reduction in pro-inflammatory cytokines after exposure to withanolides [190]. Some clinical trials report reductions in cortisol and stress markers, consistent with beneficial effects on stress-sensitive pathways, but direct measurements of BDNF in humans are still limited [191].

Another emerging plant, *Centella asiatica*, has been studied in animal models because it increases the expression of BDNF and its TrkB receptor in the hippocampus of rats subjected to stress. It also showed potential for inhibiting the production of inducible nitric oxide synthase (iNOS) and cyclooxygenase-2 (COX-2), thus preventing oxidative stress [192]. Furthermore, a study pointed to an increase in BDNF in the prefrontal cortex after the administration of *Centella asiatica* in animal models [193].

### 4.4. Modulation of the HPA Axis

The HPA axis is an essential modulator of responses to changes observed in MDD. The plant *Hypericum perforatum* and its compounds have been shown to modulate the responses of this axis in studies with animals induced to stress after 8 weeks of treatment by reducing corticosterone secretion and decreasing the release of adrenocorticotropic hormone (ACTH) [84].

In one study, the plant *Rhodiola rosea*, together with its constituents, was shown to decrease serum corticosterone levels and limit hypothalamic activation, which evidenced its connection with endocrine hormones [88]. Another study using animal stress models showed elevated levels of messenger ribonucleic acid (mRNA) and CRH in the hypothalamus in the group subjected to stress, and treatment reversed these levels. In addition, the plant also managed to reduce ACTH levels [194].

The *Melissa officinalis* plant associates its therapeutic potential with the rosmarinic acid compound. In a study with rats under stressful conditions, the compound had effects on GR levels [195]. In a study with mice, after 21 days of corticosteroid injections, ACTH and CRH levels increased in the model group, suggesting hyperactivation of the HPA axis. Treatment with rosmarinic acid, on the other hand, reduced the levels of these hormones in mice [179].

*Passiflora incarnata* is rarely mentioned in research on its potential effects on the HPA axis; however, some studies suggest that other variables of the plant may have effects, such as *Passiflora caerulea*, which has been shown to reduce stress levels in animal models [196]. There is also limited information on the relationship between the plant *Valeriana officinalis* and the HPA axis, although its connection with GABAergic neurons has been demonstrated [197].

The use of *Cannabis sativa* has been studied with an emphasis on the endocannabinoid system established in the brain. In humans, the plant has been shown to have activating effects on the HPA axis. In research, increased cortisol levels were found in users compared to non-users [198]. It has also been shown that the plant’s phytoconstituents act as negative feedback effectors mediated by glucocorticoids [199]. One study pointed to an increase in ACTH and corticosterone in the serum of adult rats after prenatal exposure to THC [200].

The bioactive compounds of *Panax ginseng*, mainly ginsenoside Rg1, are described in the literature as having the potential to modulate the HPA axis. In a study conducted with mice, which investigated the effect of the compound as a sleep modulator, where they received doses of Rg1 intraperitoneally, it was found that there was a decrease in proteins such as CRH in the hypothalamic paraventricular nucleus, which confirmed that Rg1 is capable of inhibiting orexin and CRH neurons [201].

In another study, *Withania somnifera* was also used in research into the HPA axis and sleep. A total of 49 male rats were divided into seven groups and subjected to eight hours of sleep deprivation per day. Four groups received medication: control group, wide platform, sleep deprivation (normal saline), and those who underwent sleep deprivation treated with 15 mg/kg, 30 mg/kg, 5.5 mg/kg, and 11 mg/kg. When the results were analyzed, sleep deprivation was found to increase the hormones corticosterone, CRH, and ACTH. In contrast, the treatments significantly reduced the levels of these hormones compared to the untreated group, even at the lowest doses [202].

*Centella asiatica* was used to investigate the antidepressant effect of its bioactive compounds in animal models. Serum corticosterone levels were evaluated and found to be elevated, which led the animals to increase their forced swimming time. With treatment based on the plant extract, it was observed that levels were reduced, which evidenced its involvement in improving HPA axis function [203].

Curcumin, a bioactive compound found in *Curcuma longa,* significantly reduces ACTH secretion by AtT20 cells in anterior pituitary cells in animal model studies using mouse tumor cells [204]. In a chronic stress model with rats, it was observed that the animals had elevated serum corticosterone levels and reduced expression of GR mRNA, which were reversed with chronic administration of curcumin, at 5 or 10 mg/kg orally [205].

## 5. Adverse Effects of Medicinal Plants for Major Depressive Disorder

Although many herbal medicines have a good safety profile, there is a risk of significant drug interactions [206]. *Hypericum perforatum* is a prominent example, inducing CYP450 enzymes that accelerate the metabolism of other drugs. These effects are primarily attributed to hyperforin [8,207,208]. A case report illustrates this risk with the worsening of the psychiatric condition of a patient using clozapine after self-medicating with *Hypericum perforatum* [207]. However, it is worth noting that an extract with low hyperforin content, Ze 117, has been shown not to cause significant pharmacokinetic changes [208].

In contrast to *Hypericum perforatum*, which acts as an enzyme inducer, *Cannabis sativa* poses a risk of drug interactions by inhibiting CYP450 enzymes [209,210]. Several in vitro and in vivo studies demonstrate that multiple cannabinoids (including CBD, THC, and some of their metabolites) potently inhibit various CYP isoforms [209,210,211,212]. Consequently, this inhibition can slow the metabolism of other drugs, increasing their blood concentrations and the risk of toxicity [211].

Other herbal medicines also interact with the CYP450 system. An in vitro study with *Withania somnifera*, for example, showed that its root extract modulated the expression and activity of CYP3A4 [213]. For *Rhodiola rosea*, the interaction profile appears complex: while the complete extract showed modest inhibition of CYP2C9 in humans [89], its constituent salidroside, when tested alone, did not present a significant risk of drug interactions via CYP450 [214].

Furthermore, herb-induced liver injury (HILI) is a focus of growing interest due to the increase in reports of hepatotoxicity. The literature points to a suspected association between the use of *Valeriana officinalis* and liver damage [215], a suspicion corroborated by a case report where the plant was the main constituent of an herbal compound [216]. Similarly, *Hypericum perforatum* also has the potential to cause hepatotoxicity [215]. An in vitro study found that hypericin may be the component responsible for the occasionally observed liver injury [217].

In line with this, studies indicate that *Melissa officinalis* also presents a potential for toxicity. Its essential oil, administered to mice (doses above 1 g/kg), caused alterations in liver and kidney functions [218]. Also, prolonged oral administration of *Melissa officinalis* extract in rats, even at low doses, induced mild to moderate lesions and altered biochemical and hematological parameters, suggesting its use is not entirely risk-free [219]. Additionally, the toxicological evaluation of *Passiflora incarnata* demonstrated that, despite low acute toxicity, subacute administration at high doses (from 1200 mg/kg) caused inflammation and impairment of liver and cardiac function in an animal model, limiting its safe use to doses below 600 mg/kg [220].

*Cannabis sativa* also fits into this context of hepatotoxic risk. An in vivo study with mice demonstrated that both high acute doses and lower, repeatedly administered doses of a CBD-enriched extract have the potential to cause liver injury [221]. Corroborating these findings, an in vitro study elucidated the mechanism underlying this risk, showing that CBD is directly cytotoxic to hepatocytes, inducing apoptosis and cell cycle arrest [222].

Beyond biochemical interactions, the clinical implications of these effects pose a significant challenge to patient safety. A primary concern is the risk of serotonin syndrome, a potentially life-threatening condition that can occur when *Hypericum perforatum* is co-administered with other serotonergic agents, such as SSRIs or SNRIs [223]. Furthermore, the potent induction of CYP3A4 by hyperforin carries critical implications for systemic drug efficacy. Notably, it is associated with contraceptive failure in patients using oral contraceptives and a dangerous reduction in the plasma concentration of anticoagulants like warfarin [86].

These risks are particularly salient in the context of polypharmacy, which is highly prevalent among patients with MDD who often manage multiple comorbidities [206]. The use of cannabinoids from *Cannabis sativa* as enzyme inhibitors further complicates this landscape, potentially elevating the serum levels of concomitant medications to toxic thresholds [99]. For these therapies to achieve translational value, clinicians must move beyond a general awareness of ‘natural’ remedies and implement rigorous screening protocols to identify and mitigate these specific clinical risks [207].

Finally, the use of herbal medicines during pregnancy for the treatment of mental disorders is common [223]. An in vitro study using placental cells suggested that, although complete extracts of *Hypericum perforatum* and *Valeriana officinalis* are likely safe at usual doses, their isolated compounds, such as hyperforin, hypericin, and valtrate, at high concentrations exhibited cytotoxic and apoptotic effects [223]. Caution with *Hypericum perforatum* is reinforced by a preclinical study that also demonstrated harm to maternal and fetal health [224]. Similarly, the concern extends to *Cannabis sativa*, where a preclinical study challenged the widespread belief in CBD’s safety by demonstrating that gestational exposure to both THC and CBD itself was associated with low birth weight and long-term neuropsychiatric changes in the offspring [225].

## 6. Translational Limitations and Regulatory Challenges

The transition of medicinal plants from successful preclinical observations to evidence-based psychiatric practice faces several multifaceted translational hurdles. A primary limitation is the lack of standardized protocols for extract optimization. The chemical complexity of these plants means that variables such as soil composition, climate, and harvest timing can lead to substantial batch-to-batch variability, compromising the reproducibility of therapeutic outcomes [9,10]. Furthermore, quality control remains a significant concern, as inconsistent concentrations of bioactive metabolites or the presence of heavy metal contaminants can impact both the pharmacological potency and the safety profile of the final product [9].

The regulatory classification of these products also presents a major barrier to widespread clinical integration. In many jurisdictions, including the USA, herbal products are categorized as dietary supplements rather than pharmaceuticals, a distinction that allows them to bypass the rigorous, phase-specific clinical trials and long-term safety monitoring required for synthetic antidepressants [63]. This regulatory ‘gray area’ creates challenges for clinicians in determining precise dosing regimens and evaluating the true risk of herb–drug interactions [206]. For instance, while products such as *Hypericum perforatum* are well documented [85], many emerging plants lack large-scale, randomized controlled trials necessary to confirm their safety as monotherapies or adjuncts in polypharmacy settings [17]. Establishing unified manufacturing standards and stricter regulatory oversight is therefore essential to fulfill the neuroprotective potential of these botanical therapies in modern psychiatric care.

## 7. Discussion and Final Considerations

The findings of this comprehensive review affirm the significant therapeutic potential of medicinal plants as alternative or adjunctive treatments for MDD. The existing literature, spanning preclinical models of stress and inflammation to controlled clinical trials, provides robust evidence supporting the antidepressant efficacy of established agents, such as *Hypericum perforatum*, and promising emerging candidates, including *Rhodiola rosea*, *Curcuma longa*, and *Withania somnifera*. *Hypericum perforatum* remains the most thoroughly validated, demonstrating efficacy in mild-to-moderate depression that is often comparable to conventional SSRIs, but with a generally more favorable side-effect profile, validating its prominent role in pharmacotherapy.

A central theme uniting the therapeutic effects of these diverse plant species is their multi-targeted neurobiological action, which offers a potential mechanistic advantage over traditional single-target antidepressants. As elucidated herein, plant bioactive compounds, including hyperforin, salidroside, curcumin, and various flavonoids, do not merely modulate monoamine reuptake; they simultaneously engage multiple pathways critical to MDD pathophysiology. These mechanisms encompass the restoration of the excitatory/inhibitory balance through GABAergic and glutamatergic systems, the enhancement of neuroplasticity (as evidenced by increased BDNF and TrkB signaling), and, crucially, the suppression of chronic neuroinflammation and oxidative stress (via the inhibition of pro-inflammatory cytokines such as IL-6 and TNF-α). This synergistic, polypharmacological approach suggests that medicinal plants may hold particular promise for patients with treatment resistance or depression subtypes characterized by elevated inflammatory markers.

Despite their therapeutic promise, significant challenges related to pharmacokinetics and safety must be rigorously addressed before the widespread clinical adoption of these therapies. PK studies reveal that many key active compounds, such as asiaticoside and curcumin, exhibit limited oral bioavailability and rapid metabolism, necessitating advanced delivery strategies, including nanoformulations or co-administration with absorption enhancers (e.g., piperine). More critically, the risk of herb–drug interactions is significant. *Hypericum perforatum* acts as a potent enzyme inducer of CYP3A4 and P-gp, while cannabinoids from *Cannabis sativa* are potent enzyme inhibitors. This bidirectional modulation of the CYP450 system means that the concomitant use of these plants with classical antidepressants, anticoagulants, or immunosuppressants requires extreme caution. Furthermore, the rising incidence of HILI associated with several plants, including *Valeriana officinalis* and high-dose *Cannabis sativa* extracts, challenges the public perception of herbal treatments as universally safe.

Moving forward, the successful integration of medicinal plants into modern psychiatric care hinges on two critical areas of development. First, future research should shift its focus from preclinical models to high-quality, standardized, and long-term randomized controlled trials, particularly for emerging plants, and include objective biomarkers such as BDNF or inflammatory markers. Second, due to the high incidence of polypharmacy in MDD patients, dedicated clinical studies are essential to determine the safety and efficacy of concomitant use of medicinal plants with classical synthetic antidepressants. By prioritizing standardized extracts, robust quality control, and a rigorous clinical and regulatory pathway, medicinal plants can fulfill their potential to contribute to a more personalized and multi-mechanistic approach to treating MDD.

Table 2 shows the medicinal plants discussed in this study and their mechanisms of action.

**Table 2:** Main medicinal plants for depression and their mechanisms of action.

Medicinal Plant	Main Compounds	Mechanisms of Action	References
*Cannabis sativa*	Cannabinoids, terpenes	Modulation of neurotransmitter signaling	[55,144]
*Centella asiatica*	Asiaticoside, madecassoside, asiatic acid, madecassic acid	Modulates neurotransmitters (serotonin, dopamine), increases BDNF/TrkB expression, and inhibits iNOS/COX-2 (reducing oxidative stress)	[80,81,107,192,193]
*Curcuma longa*	Curcumin	Increased levels of BDNF and serotonin, possibly acting through modulation of NMDA and 5-HT7A-mediated pathways	[70,71]
*Hypericum perforatum*	Hypericin, hyperforin, flavonoids	Reuptake inhibition of serotonin, norepinephrine, dopamine, and glutamate	[12]
*Melissa officinalis*	Triterpenoids, volatile compounds, flavonoids, and phenolic acids	Increases serum BDNF concentration	[25,26]
*Panax ginseng*	Ginsenoside	It acts on the dopaminergic and serotonergic systems, modulating BDNF expression	[77]
*Passiflora incarnata*	Polyphenols, carotenoids, indole alkaloids	Modulation of neurotransmitter signaling	[34,139,141]
*Rhodiola rosea*	Rhodioloside, salidroside	Improve 5-HT level, induce neural stem cell proliferation, decrease pro-inflammatory cytokine levels, and increase BDNF	[21,22]
*Valeriana officinalis*	Valerenic acid, flavonoids, caffeoylshinic acid	Modulation of the GABA system	[130,226]
*Withania somnifera*	Withanolides	Modulates inflammatory and biochemical markers and increases BDNF and serotonin levels	[74,76]

In summary, this review demonstrates that several medicinal plants exhibit therapeutic potential for the management of MDD. While neurobiological mechanisms are well supported by animal models, current clinical research indicates significant antidepressant effects in humans, particularly for species such as *Hypericum perforatum* and *Curcuma longa*. However, the definitive translation into psychiatric practice remains incomplete.

Current clinical evidence is constrained by the heterogeneity of formulations and by the lack of large-sample, long-term randomized controlled trials. Additionally, the lack of standardization of dosages and extracts makes direct comparisons across interventions challenging. Therefore, while medicinal plants represent a promising frontier, strengthening evidence through more rigorous, standardized clinical protocols is essential to ensure their safety and efficacy as alternatives or adjuncts to conventional treatments.

Table 3 compares the available preclinical and clinical evidence for the plants, highlighting the experimental data and its limitations.

## Figures and Tables

**Table 1:** Comparative pharmacokinetic profile of major bioactive compounds and extracts from medicinal plants with antidepressant potential.

Medical Plants	Compound/Extract	Model	Cmax	Tmax	Half-Life (t½)	References
*Hypericum perforatum*	Hypericin/Hyperforin	Human	8–15 ng/mL	4.5 h	17.5 h	[118]
*Rhodiola rosea*	Salidroside	Rat	2.5 µg/mL	0.5 h	1.1 h	[119]
*Melissa officinalis*	Rosmarinic acid	Human	1.8 µg/mL	0.7 h	1.5 h	[120]
*Passiflora incarnata*	Flavonoid-rich extract	NA	NA	NA	NA	[121]
*Valeriana officinalis*	Valerenic acid	Rat	0.8 ng/mL	2 h	5 h	[122]
*Cannabis sativa*	Cannabidiol (CBD)	Human	114 ng/mL	3 h	18–32 h	[123]
*Panax ginseng*	Ginsenosides	Human	10–50 ng/mL	2–6 h	7–15 h	[124]
*Withania somnifera*	Withanolides	Rat	1.2 µg/mL	0.6 h	0.6 h	[125]
*Centella asiatica*	Madecassoside/Asiaticoside	Rat	2.0 µg/mL	0.3 h	0.3 h	[108]
*Curcuma longa*	Curcumin	Human	0.5–2.0 µM	1–2 h	1–2 h	[111]

NA—not applicable.

**Table 3:** Summary of preclinical and clinical evidence for medicinal plants with antidepressant potential.

Medical Plants	Preclinical Evidence	Clinical Evidence	Clinical Sample Size (n)	Main Results	Limitations	References
*Hypericum perforatum*	Tests in rodent models; inhibition of monoamine reuptake.	Several randomized clinical trials and meta-analyses	*N* > 2000	A reduction in scores on the Hamilton Depression Rating Scale, similar to that observed with SSRIs	Drug interactions, variable extracts	[15,82]
*Rhodiola rosea*	Chronic stress models; increased BDNF expression	Small clinical trials	*N* ~ 60–120	Improved depressive symptoms	Small samples, short duration	[135]
*Melissa officinalis*	Rodent anxiety/depression models	There are no robust randomized clinical trials	-	-	Lack of clinical trials	[227]
*Passiflora incarnata*	Animal models of anxiety/depression	Limited clinical studies (not specific for MDD)	*N* < 50	Anxiolytic effects	Weak evidence for depression	[228]
*Valeriana officinalis*	GABAergic modulation in rodents	There are no robust randomized clinical trials	-	-	Mainly studied for sleep disorders	[229]
*Cannabis sativa*	Rodent depression models; anti-inflammatory effects	Observational studies and small clinical trials	Variable	Mixed antidepressant outcome	Regulatory and methodological limitations	[230,231]
*Panax ginseng*	Chronic stress rodent models	Small clinical trials	*N* ~ 40–100	Mood improvement	Not MDD-specific, heterogeneous outcomes	[232,233]
*Withania somnifera*	Stress-induced rodent models	Small clinical trials	*N* ~ 60–80	Reduced stress and depression scores	Short follow-up, mixed populations	[234,235]
*Centella asiatica*	Neuroinflammation and CUMS models	No randomized clinical trials were found.	-	-	Only preclinical evidence	[236]
*Curcuma longa*	Rodent depression models	Meta-analyses of randomized clinical trials	*N* > 1000	Reduced depressive symptoms	Bioavailability issues	[82,237]

## Data Availability

No new data were created or analyzed in this study.

## Abbreviations

5-HT	5-hydroxytryptamine
7-COOH-CBD	7-carboxycannabidiol
7-OH-CBD	7-hydroxycannabidiol
Aβ	beta-amyloid
AcOEt	ethyl acetate
ACTH	adrenocorticotropic hormone
AE	aqueous extract
Akt	protein kinase B
AUC	area under the plasma concentration curve
BBB	blood–brain barrier
BDI	Beck Depression Inventory
BDNF	brain-derived neurotrophic factor
BuOH	butanol
CBD	cannabidiol
CGI	Clinical Global Impression
CMS	chronic mild stress
CNS	central nervous system
COX-2	cyclooxygenase-2
CPZ	chlorpromazine
CREB	cAMP response element-binding protein
CRH	corticotropin-releasing hormone
CYP	cytochrome P450
Cmax	maximum concentration reached
FAAH	fatty acid amide hydrolase
FST	forced swim test
GABA	gamma-aminobutyric acid
GLU	glutamate
GPx	glutathione peroxidase
GR	glucocorticoid receptor
GSK-3β	glycogen synthase kinase-3beta
HAM-D	Hamilton Depression Rating Scale
HE	ethanol extract
HILI	herb-induced liver injury
HO-1	heme oxygenase-1
HPA	hypothalamic–pituitary–adrenal
Hp-C	conventional *Hypericum perforatum*
Hp-MF	multi-fractionated *Hypericum perforatum*
ICAM-1	intercellular adhesion molecule-1
IL-1β	interleukin-1 beta
IL-6	interleukin-6
iNOS	inducible nitric oxide synthase
LPS	lipopolysaccharide
LTP	long-term potentiation
MAGL	monoacylglycerol lipase
MAO	monoamine oxidase
MAPK	mitogen-activated protein kinase
MDA	malondialdehyde
MDD	Major Depressive Disorder
mRNA	messenger ribonucleic acid
NADPH	nicotinamide adenine dinucleotide phosphate
NF-κB	nuclear factor-kappa B
NLRP3	NOD-like receptor family pyrin domain containing 3
NMDA	N-Methyl-D-Aspartate
Nrf2	nuclear factor erythroid 2-related factor 2
NA	Not available
OBX	olfactory bulbectomy
P-gp	P-glycoprotein
PSD-95	postsynaptic density protein 95
ROS	reactive oxygen species
SJW	St. John’s wort
SOD	superoxide dismutase
SSRIs	selective serotonin reuptake inhibitors
SYP	synaptophysin
THC	Δ-9-tetrahydrocannabinol
Tmax	time to maximum concentration
TNF-α	tumor necrosis factor-alpha
TrkB	tropomyosin receptor kinase B
TST	tail suspension test
t½	elimination half-life
UHPLC-MS/MS	ultra-high-pressure liquid chromatography with mass spectrometry
UGTs	uridine 5′-diphospho-glucuronosyltransferases
USA	United States of America
VCAM-1	vascular cell adhesion molecule-1
